# Impact of *Helicobacter pylori* and metabolic syndrome-related mast cell activation on cardiovascular diseases

**DOI:** 10.3389/fgstr.2024.1331330

**Published:** 2024-03-04

**Authors:** Michael Doulberis, Apostolis Papaefthymiou, Stergios A. Polyzos, Marina Boziki, Evangelos Kazakos, Maria Tzitiridou-Chatzopoulou, Elisabeth Vardaka, Carola Hammrich, Hasan Kulaksiz, Daniele Riva, Christos Kiosses, Ioannis Linas, Maria Touloumtzi, Aggeliki Stogianni, Jannis Kountouras

**Affiliations:** ^1^ Private Gastroenterological Practice, Gastroklinik, Gastroklinik, Horgen, Switzerland; ^2^ Division of Gastroenterology and Hepatology, Medical University Department, Kantonsspital Aarau, Aarau, Switzerland; ^3^ Second Medical Clinic, School of Medicine, Aristotle University of Thessaloniki, Ippokration Hospital, Thessaloniki, Greece; ^4^ Department of Gastroenterology, University Hospital of Larisa, Larissa, Greece; ^5^ First Laboratory of Pharmacology, Aristotle University of Thessaloniki, Thessaloniki, Greece; ^6^ Pancreaticobiliary Medicine Unit, University College London Hospitals (UCLH), London, United Kingdom; ^7^ Second Neurological Department, Aristotle University of Thessaloniki, AHEPA University General Hospital of Thessaloniki, Thessaloniki, Greece; ^8^ School of Healthcare Sciences, Midwifery Department, University of West Macedonia, Kozani, Greece; ^9^ Department of Nutritional Sciences and Dietetics, School of Health Sciences, International Hellenic University, Macedonia, Greece; ^10^ School of Dental Medicine Georg-August University, Göttingen, Germany; ^11^ Private Gastroenterological Practice, Gastrocentro Plus, Lugano, Switzerland; ^12^ Private Gastroenterology Practice, Praxis Gastroenterologische Gruppenpraxis (GGP), Bern, Switzerland; ^13^ Department of Radiology, Ippokration Hospital, Thessaloniki, Greece

**Keywords:** *Helicobacter pylori*, mast cells, vascular, metabolic syndrome, atherosclerosis

## Abstract

*Helicobacter pylori*, a widely renowned bacterium, has recently gained attention owing to its potential impact on extragastric health. The emergence of research linking *H. pylori* infection with metabolic syndrome (MetS)-related cardiovascular diseases (CVDs) has raised intriguing questions about the pathogenic linkage and its translational implications for clinicians. MetS encompasses a collection of metabolic abnormalities that considerably elevate the risk of CVDs and cerebrovascular diseases. Emerging evidence supports a potential pathogenetic role of *H. pylori* for MetS-related disorders through mechanisms implicating chronic smoldering inflammation, insulin resistance (IR), and modulation of immune responses. One intriguing aspect of this possible connection is the role of mast cells (MCs), a subset of immune cells representing innate immune system effector cells. They play a fundamental role in innate immune responses and the modulation of adaptive immunity. Activated MCs are commonly found in patients with MetS-related CVD. Recent studies have also suggested that *H. pylori* infection may activate MCs, triggering the release of pro-inflammatory mediators that contribute to IR and atherosclerosis. Understanding these intricate interactions at the cellular level provides new insights into the development of therapeutic strategies targeting both *H. pylori* infection and MetS-related MCs activation. This review investigates the current state of research regarding the potential impact of *H. pylori* infection and MetS-related MCs activation on the pathophysiology of CVD, thereby opening up new avenues for related research and paving the way for innovative approaches to prevention and treatment in clinical practice

## 1 Introduction

*Helicobacter pylori* infection, representing a high worldwide burden (1), has recently garnered growing attention for its potential role in various systemic disorders including cardiovascular diseases (CVDs) (2–7). Although *H. pylori* has been primarily associated with gastric disorders, particularly peptic ulcers, gastric adenocarcinoma, and MALT (mucosa-associated lymphoid tissue) lymphoma (8, 9), mounting evidence suggests that this bacterium can exert a systemic influence, affecting diverse pathophysiological processes beyond the gastrointestinal tract (2, 6, 7). The intricate relationship of *H. pylori* with the immune system and its potential for inducing chronic low-grade (“smoldering”) inflammation in remote organs have raised intriguing questions about its role in extragastric diseases.

Metabolic syndrome (MetS) constitutes a cluster of diverse metabolic risk factors that lead to a clinical syndrome. Despite the plethora of different definitions and cutoffs, MetS prerequisites the presence of the following conditions: abdominal obesity, insulin resistance (IR), type 2 diabetes mellitus (T2DM), dyslipidemia, nonalcoholic fatty liver disease (NAFLD), arterial hypertension (AH), and CVD (10).

In this respect, an emerging body of evidence supports a connection between MetS and active *H. pylori* infection: both pathologies appear to promote the pathogenesis of each other (11). *H. pylori* infection is positively associated with MetS (12), and its eradication affects MetS parameters (13). Moreover, *H. py*lori serves as an independent risk factor for CVD, and its eradication is safe from a cardiovascular perspective (14). The results of a recent meta-analysis also indicated a positive correlation between *H. py*lori infection and MetS and IR (15). A further piece of scientific evidence supports the suggestion that *H. pylori* infection is an independent risk factor for MetS-related NAFLD and is correlated with an increased degree of steatosis (16). Moreover, active *H. pylori* infection has been found to be independently positively associated with the severity of MetS-related non-alcoholic steatohepatitis (NASH) and other MetS-related parameters including IR, dyslipidemia, and AH (17). This association also extends to the context of pregnancy (18). In this regard, active *H. pylori* infection is equally associated with MetS-related systemic pathologies, especially cardio-cerebrovascular diseases (C-CVDs), the end point of MetS (19–21).

Mast cells (MCs), a subgroup of immune cells first detected to mediate allergic and anaphylactic reactions, represent innate immune system effector cells playing an important role in innate immune reactions and in modulating adaptive immunity (22).

Apart from local effects, some more recent data regarding the activation of MCs in *H. pylori* infection and/or MetS also concentrated on systemic effects. For instance, several infections, mostly *H. pylori* infection, have been associated with chronic urticaria (23). Many autoimmune disorders such as systemic lupus erythematosus, polymyositis, dermatomyositis, and rheumatoid arthritis have been connected with chronic urticaria (23). In this regard, *H. pylori* infection and/or MetS are linked to such disorders as systemic lupus erythematosus and rheumatoid arthritis (24–27). MCs also contribute to the pathophysiology of these autoimmune disorders (26, 28). Moreover, recent data have indicated the involvement of MCs in the pathophysiology of additional *H. pylori* and MetS-associated disorders, such as NAFLD (16, 17, 22). NAFLD is strongly associated with a higher risk of systemic disorders including C-CVD and neuroautoimmune pathologies, the end points of MetS (22, 29–31). In the same way, for instance, MCs induce dyslipidemia, atherosclerosis, and/or AH (32–34). All of these disorders, also connected with *H. pylori* infection and MetS, are risk factors for C-CVD and neurogenerative pathologies (21, 35–37). As another final example, *H. pylori*-induced vacuolating cytotoxin A (VacA) exhibits chemotactic activities toward bone marrow-derived MCs (BMD-MCs) and induces them to produce pro-inflammatory cytokines involved in topical pathologies. BMD-MCs, beyond the gastrointestinal tract, also reside adjacent to blood and lymphatic channels, predominantly under epithelial surfaces including the blood–brain barrier. MCs can be activated by the corticotropin-releasing hormone, secreted under stress, to induce mediators including chymase or tryptase, interleukin 8 (IL-8), and vascular endothelial growth factor, which disrupt the blood–brain barrier, leading to neurodegenerative pathologies (38). MCs activation, by mediating blood–brain barrier damage, contributes to cognitive impairment (39). Likewise, *H. pylori* VacA promotes the intracellular survival of *H. pylori*, and activated monocytes (possibly infected with *H. pylori* owing to defective autophagy) could access the brain (Trojan horse theory) through blood–brain barrier damage, thereby leading to neurodegeneration (6).

Specifically, concerning MCs-related pathogen internalization, related data indicated that *Staphylococcus aureus* (S. Aureus) is able to internalize within BMD-MCs (40) in a subterfuge manner, where it evades detection and elimination. Live *S. aureus* can be found viable up to 5 days later within MCs (41). The interactions between MCs and *S. aureus*, including the internalization procedure, demonstrated a noticeable role of staphylococcal enterotoxin B in stimulating the uptake of the bacteria into MCs (42). In contrast to other bacterial pathogens, *S. aureus* is able to internalize into MCs and could induce a unique response. There is a crosstalk between the internalized *S. aureus* and human MCs, and a fraction of the internalized *S. aureus* undergoes a profound transcriptional reprogramming within human MCs to adapt to the nutrients and stress encountered in the intracellular environment and remain viable (43). Of note is that MCs can act as professional phagocytes in the pathophysiology of biofilm-related disorders (44).

Since there is currently no scientifically established evidence in the literature regarding the possible capability of *H. pylori* to internalize MCs and evade their elimination, further investigation is required to elucidate this field.

Recent research has unveiled their implication in non-allergic and/or non-anaphylactic inflammatory pathologies, including MetS-related disorders such as NAFLD (45), the hepatic component of MetS having been recently renamed as metabolic (dysfunction)-associated fatty liver disease (MAFLD). MAFLD exhibits a 10-year high risk of atherosclerotic CVD, and it is strongly associated with a higher risk of systemic conditions including C-CVD and neuroautoimmune pathologies, the end points of MetS-linked MAFLD. Innate immune cell activation including MCs could contribute to the pathophysiology of MAFLD and its complications; therefore, restricting the activation of MCs may represent a possible novel therapeutic option for MAFLD and its complications (22, 46). Likewise, recent evidence has indicated that trained innate immunity or trained immunity can also lead to chronic inflammatory pathologies and, thus, to miscellaneous MetS-related disorders, such as atherosclerotic CVD (46). The training of innate immune cells, such as monocytes, macrophages, natural killer cells, and MCs, is possibly involved in the pathophysiology of MAFLD and its complications (46–48), and MCs may be involved in systemic MetS-connected inflammatory pathologies through several mechanisms (38, 49–51).

Apart from the implication of MCs in MetS-related disorders, their direct activation by *H. pylori* infection may also be implicated in *H. pylori*-related pathologies (52, 53). In this regard, at the topical level, the interactions between MCs and *H. pylori* appear to contribute to the pathophysiology of gastritis (53), and interactions among *H. pylori*, gastric epithelium, and MCs through inflammatory cytokine induction contribute to *H. pylori*-related gastric pathologies. While *H. pylori* can be eradicated by oral antibiotic administration in most patients, it is worth noting that chronic gastritis may persist even after successful *H. pylori* eradication regiments, particularly in the presence of chronic gastritis-related dysplasia (54, 55). Thus, it is beneficial to clinical prognosis when treatments can manage the underlying inflammatory process through several possible therapeutic targets, including MCs (56). At the systemic level, *H. pylori*-related stimulation of the innate immune cells including MCs may be involved in the aforementioned systemic MetS-related inflammatory pathologies through several mechanisms (38). Similarly, *H. pylori* infection may also be connected with trained immunity and, by provoking gut dysbiosis (57), may play a role in MALFD and other MetS-related CVD through several mechanisms (46). Repeated inflammation owing to *H. pylori* infection appears to be sufficient to induce trained immunity (58).

Furthermore, MCs activation may induce an augmented gut permeability, allowing potentially harmful gut symbiotic molecules to spread into circulation and contribute to systemic pathologies (59, 60).

Thus, *H. pylori* infection and MetS-related MCs involvement in the pathophysiology of topical and systemic pathologies may offer benefits through the application of novel related strategies toward increasing the eradication of *H. pylori*.

In view of the aforementioned considerations, we aimed to investigate the hypothesis of the potential pathogenetic link between *H. pylori* infection and the activation of MCs in the context of MetS with special focus on CVDs. Despite this connection seeming intricate and multifaceted, elucidating it could yield significant clinical insights. If substantiated, this hypothesis could provide clinicians with a novel perspective on the etiology and management of CVDs associated with *H. pylori*-related MetS-linked MCs activation. We will explore the current knowledge of each component of this hypothesis, aiming at shedding light on the intricate web of interactions that may culminate in CVDs. We will also discuss the potential translational implications for medical practitioners and the avenues for future research.

## 2 *H. pylori* and MetS-related CVD

Both *H. pylori* infection and MetS are highly prevalent worldwide. The mean global prevalence of *H. pylori* infection is 58%, partly owing to immigrants coming from nations with a high prevalence of *H. pylori* infection (61). The pooled prevalence estimate for MetS is 24%, and the prevalence rate of its components including being overweight/obese is from 35.6% to 44.1% (62, 63). There is growing evidence (**Table 1**) for a potential association between *H. pylori* infection and IR syndrome or MetS and its related morbidities, including abdominal obesity, T2DM, dyslipidemia, AH, MAFLD, and CVD (3). In particular, *H. pylori* infection contributes to IR, the central feature of MetS and a crucial component in the pathogenesis of atherosclerosis and AH-induced target tissue injuries.

**Table 1:** Summary of the most important molecules (mediators) implicating in the possible interaction among *Helicobacter pylori* infection, mast cells, and cardiovascular disease.

Molecule (mediator)	Suggested pathogenesis
IL-33, TNF-α	Implicated in MCs activation as a result of *H. pylori* infection and may promote atherosclerosis
Contents of MCs secretory granules (histamine, proteases, serotonin, dopamine), TNF-α, IL-4, and IL-5, and growth factors (stem cell- and fibroblast growth factor)	Implicated in MCs activation as a result of *H. pylori* infection and may promote atherosclerosis
CCL2	Its expression is induced by leptin, the latter of which is controlled by MCs. CCL2 is associated with atherosclerotic lesions and their lipid deposition and is one of the major chemokines being involved in the development and progression of CVD
TNF-α, IL-6	*H. pylori* associated TNF-α and IL-6 play important roles in the regulation of the synthesis of other acute phase proteins, which are recognized risk factors for MetS-connected atherosclerosis. The same molecules exert chemotactic actions on MCs
Galectin‐3	Galectin-3 is connected with severe CVD outcomes and all‐cause mortality in MetS-related type 2 diabetes mellitus independently of conventional risk factors. Galectin-3 is a member of the β-galactoside-binding lectin family expressed among other, in MCs and it also binds to *H. pylori* O-antigen
LPS	*H. pylori*-related LPS demonstrates pro-inflammatory properties and contributes to atherosclerosis and CVD. Of note, LPS also improves FcepsilonRI-mediated MCs degranulation
VEGF, IL-8, chymase and tryptase	Linkage to *H. pylori* infection: *H. pylori* stimulates MCs directly or via gastrin induction and MCs are actively involved in the pathogenesis of *H. pylori*-associated pathologies, including CVD

CVD, cardiovascular diseases; *H. pylori*, *Helicobacter pylori*; IL, Interleukin; LPS, Lipopolysaccharide; MCs, mast cells; MetS; metabolic syndrome; TNF, tumor necrosis factor; VEGF, vascular endothelial growth factor.

To colonize the human gastric chamber, *H. pylori* first needs to overcome two essential physical barriers: stomach acid and the gastric mucous layer. The low-pH environment in the human stomach plays a fundamental role in preventing bacterial growth and colonization. *H. pylori* urease is thought to buffer the acid in the gastric lumen through the hydrolysis of urea to bicarbonate and ammonia, thereby inducing a neutral microenvironment around the bacterium (64, 65). The activity of urease is coupled with an acid-gated urea channel, which enables *H. pylori* to maintain a neutral cytoplasmic pH when exposed to acidic conditions (66). *H. pylori* bacteria that have survived the acid in the stomach are then able to negotiate the thick gastric mucous layer, courtesy of their spiral shape and multiple polar flagella (67).

After traversing the mucous layer and reaching the gastric epithelium, *H. pylori* avoids being flushed away during the continual replenishment of the gastric mucosa by using a range of adhesins to tightly attach to the cells of the gastric epithelium (68). This allows *H. pylori* to escape the acidic conditions in the lumen of the stomach and gain access to gastric epithelial cells, which could act as a source of essential nutrients following VacA-dependent cell permeabilization (69), thereby leading to its long-term persistency in the gastric mucosa and, thus, triggering chronic inflammation.

In addition, is well known that a direct interaction between *H. pylori* and MCs takes place in the gastric mucosa niche. Specifically, the bacterial ecosystem of the gastric niche is significantly affected by *H*. *pylori* infection (70), which may contribute to the broad spectrum of clinical manifestations in chronically infected patients. The natural niche for *H. py*lori is the human stomach, and *H. pylori* has developed a unique set of virulence factors that allow its survival in this unique ecological niche, the human stomach (71).

The interaction between *H. pylori* and MCs is associated with a significant increase in the number of activated MCs into the extracellular matrix of the gastric mucosa niche (53). The inflammatory process during *H. pylori* infection within the gastric mucosa niche involves the interactions between *H. pylori*, the gastric epithelium, and MCs *via* pro-inflammatory cytokines including IL-33 and tumor necrosis factor alpha (TNF-α) (56). Such cytokines, acting at a distance, are involved in the pathophysiology of atherosclerotic CVD (72–75). Similarly, in *H. pylori*-associated chronic gastritis, the density, and particularly the number of gastric niche MCs-related epithelial cells, is correlated with a high number of apoptotic epithelial cells (76).

Beyond the gastric mucosa niche, the oral cavity is another niche for *H. pylori* and could be the trigger of *H. pylori* infection, re-infection, and transmission into the stomach (77). Since MCs activation is also linked to various inflammatory conditions of the oral cavity (78), further research is warranted to elucidate the possible direct interaction between *H. pylori* and MCs taking place in the oral cavity niche, with a potential relation to CVDs.

In the context of CVDs, persistent *H. pylori* infection stimulates both local and systemic immune responses involved in the pathophysiology of atherosclerotic CVDs (79–81).

*H. pylori* infection is linked to arterial stiffness, an early indicator of atherosclerosis associated with MetS-related AH and an independent predictor of CVD complications and all-cause fatality (21). Interestingly, the occurrence of *H. pylori* DNA in atherosclerotic plaques and the *H. pylori* infection-related carotid plaque onset in adults without previous CVD suggest that this bacterium could play a role in the pathogenesis of atherosclerosis and that its eradication may decrease the burden of MetS-related atherosclerosis (82–84). *H. pylori* infection has been associated with fatal MetS-related ischemic heart disease and stroke outcomes than with nonfatal events ascertained by general practitioners. *H. pylori* infection is independently related to the future risk of CVD death; the assessed 10-year CVD risk increases as the grade of *H. pylori*-related gastritis increases. *H. pylori*-related galectin-3, which is positively associated with MetS, beyond MAFLD, is also connected to a higher risk of all-cause mortality and particularly with heart failure and C-CVD mortality (85). *H. pylori*-associated galectin-3 upregulation and MetS appear to be implicated in the persistent and progressive dysfunction of several organs including the liver, CVD, kidney, and the brain (86).

Focusing mainly on CVD, for instance, galectin-3 is associated with severe CVD outcomes and all-cause mortality in MetS-related T2DM independently of conventional risk factors (87). Acute myocardial infarction, a potentially fatal CVD complication, is closely linked to MetS (3), and *H. pylori* is a risk factor for potential lethal acute coronary syndrome including acute myocardial infarction. *H. pylori* infection also appears to provoke platelet activation/aggregation and an increment in various proatherogenic factors, such as MetS-related homocysteine, thereby contributing to *H. pylori*-associated CVD risk (88–90). Moreover, interactions between *H. pylori* infection and MetS-related hyperhomocysteinemia appear to contribute to atherosclerosis linked to systemic pathologies, including C-CVD (91).

Atrial fibrillation (AF) is an important contributor to CVD morbidity and mortality mainly due to AF-induced strokes. In this respect, *H. pylori*-related MetS could contribute to the development and/or progression of CVD, including AF, *via* several potential mechanisms including the pro-inflammatory cytokines TNF-α and IL-6 involved in atherosclerosis and AF. Similarly, IR is associated with AF and a twofold increase in CVD outcomes, including stroke (92). Active *H. pylori* infection-related MetS might also contribute to the pathophysiology of MAFLD and its related AF complications. Therefore, eradicating this bacterium may offer protection against the aforementioned pathologies; thus, further investigation is also needed (92, 93).

It is important to note that inflammatory pathways, including the nuclear factor kappa B (NF-κB) signaling cascade, are activated in response to the presence of *H. pylori* (37, 94). This inflammation can disrupt insulin signaling and contribute to the aforementioned IR (37, 95). Furthermore, *H. pylori*-related lipopolysaccharide (LPS) has demonstrated pro-inflammatory properties and contributes to atherosclerosis and CVD. The gut bacteria, including *H. pylori*, can initiate the inflammatory state of obesity and IR *via* the activity of LPS, and there is strong association between high LPS activity and the prevalence of MetS. For instance, the plasma LPS levels were reported to be 76% higher in patients with T2DM than in the control group (96). In this respect, *H. pylori* infection has been associated with alterations in the gut microbiota composition (5, 97, 98), and *H. pylori*-related MetS microbiota dysbiosis could play a role in the pathogenesis of MAFLD (17) and its systemic complications such as stroke. In contrast, fecal microbiota transplantation may prevent MAFLD and regulate the brain injury-induced dysbiosis, thereby improving stroke outcomes (99, 100).

Moreover, MetS is linked to dyslipidemia, and *H. pylori* infection can induce the host’s abnormal lipid metabolism, including lipoprotein (a), low-density lipoprotein cholesterol (LDL-C), high-density lipoprotein cholesterol (HDL-C), and total cholesterol (TC). *H. pylori*-related lower HDL-C can induce dyslipidemia, whereas *H. pylori* eradication significantly reduces TC, LDL-C, and fibrinogen, an independent risk factor for MetS-related CVD, but increases HDL-C. Furthermore, beyond dyslipidemia, *H. pylori* eradication improves the other MetS-related components, such as AH, IR, body mass index, and total oxidant status. Thus, *H. pylori* infection might serve as risk factor for MetS-related components, including dyslipidemia; thereby, its eradication possibly prevents the prevalence of MetS-related CVD (20).

Finally, *H. pylori* infection has been linked to alterations in appetite-regulating hormones. Ghrelin, an orexigenic hormone that stimulates hunger, is raised in individuals with *H. pylori* infection (101) and could contribute to increased food intake and obesity, both of which are central components of MetS. Although the linkage between *H. pylori* and MetS is evident, it is necessary to emphasize the role of confounding factors. Lifestyle factors, including diet and physical activity, play a pivotal role in the onset of MetS. These factors can also affect the rates of *H. pylori* infection and the course of disease (102). Therefore, unraveling the direct effects of *H. pylori* from lifestyle-related factors remains a challenge.

Apart from *H. pylori*, the rest of the gut microbiota is thought to be implicated in the pathobiology of CVD patients. Particularly, evidence remarkably supports an active crosstalk between CVDs and gut microbiota disturbances (dysbiosis) related to intestinal inflammation, while the stability of the gut microbiota exhibits a repressive effect on the development and progression of CVDs (103). Specifically, available evidence indicates a close connection between gut dysbiosis and the increased risk of cardiometabolic disorders and CVDs. Detrimental changes in the gut microbiota composition are associated with intestinal inflammatory reactions that lead to adverse remodeling of the host phenotype, predisposing to several pathological conditions such as obesity, IR, atherosclerosis, and associated disorders, ultimately leading to CVDs (104). Recent data indeed indicate that gut dysbiosis is related to chronic intestinal inflammation and MetS-related disorders inducing CVD, thereby appearing to be a promising target for the therapeutic management of these diseases (105). Gut dysbiosis may influence the cardiometabolic risk and CVD development either directly, *via* metabolite production, or indirectly, by interacting with the immune system (106). Gut dysbiosis triggers both the local intestinal inflammation involved in the pathophysiology of local disorders, including irritable bowel syndrome (IBS) and inflammatory bowel disease (IBD) (107), and systemic inflammation, contributing to the development and progression of MetS-related disorders including CVD (108).

In this respect, there is a relationship between *H. pylori* and the gut microbiota. Colonization of *H. pylori* in the gastric mucosa changes not only the local ecosystem but also the composition of the intestinal tract microbiota, consequently modulating the concentrations of microbial metabolites that can be introduced as disease markers (57). *H. pylori* infection induces profound alterations and increases the diversity in the human microbiota (109), and there is an interplay between *H. pylori* infection, gut dysbiosis, and extragastric disorders, particularly atherosclerotic CVD (110). Furthermore, MCs is associated with gut dysbiosis-related disorders such as IBD (111–113) and IBS (114).

Taken together, there is a potential crosstalk between CVD and gut dysbiosis related to *H. pylori* and MCs. Further research is warranted to elucidate in depth this critical topic offering significant clinical implications.

## 3 Role of MetS and *H. pylori* infection in the pathophysiology of MCs activation

MCs, originally renowned for their involvement in allergic and anaphylactic reactions, have emerged as pivotal players in the intricate web of MetS pathogenesis (115, 116). They were initially described by Waldeyer and later on by his student Ehrlich, who gave them their name (Mastzellen, namely, well fed, due to their high amount of cytoplasmatic granules) (51). Of note is that MCs are not found in the bloodstream as mature forms; instead, they end up in the epithelial and connective tissues of target organs and they reside there, where they undertake their final maturation, i.e., differentiation. MCs are multifunctional tissue-specific innate immune cells located in sites throughout the body, including the adipose tissue (117) and the heart (118). The topography of the adipose tissue places them at the forefront of the obesity-driven chronic inflammation that characterizes MetS (119). In this tissue, the activation of MCs chemotactically triggers the recruitment of immune cells, such as lipid-uptaking foamy macrophages, further amplifying the inflammatory milieu and angiogenesis (120, 121). This immune cell infiltration is a hallmark of IR adipose tissue and plays a crucial role in perpetuating the MetS phenotype (122, 123).

In the human heart, MCs are mainly distributed in the myocardial tissue, adjacent to capillaries and venules (124), and MCs degranulation, an early phase of their activation (125), occurs not only in the context of allergy but also in other pathologies including cardiac injury (126). Their secretory granules contain a plethora of vasoactive mediators including excessive specific substances (e.g., histamine), proteases (e.g., tryptase and chymase), non-MCs-specific proteases (cathepsin G), amines (serotonin and dopamine), cytokines (TNF-α, IL-4, and IL-5), and growth factors [stem cell factor (SCF) and fibroblast growth factor (FGF)] (127).

Regarding MetS-related MCs activation (51), the aforementioned mediators contribute to the onset of IR by compromising the insulin signaling pathways (51), which is associated with MetS-related atherosclerosis (128). The relationship between atherosclerosis and MetS is multifactorial, with obesity, dyslipidemia, and IR playing substantial roles in both its onset and progression (129).

In this regard, MCs have been associated with the dysregulation of MetS-related adipokines (130, 131), namely, bioactive molecules secreted directly by adipocytes. Leptin, an adipokine that orchestrates appetite and energy expenditure, is known to be increased in obesity (132). Higher concentrations of circulating leptin are linked to the augmented severity of MAFLD (130). In contrast, MetS-related adiponectin ameliorates liver histology in MAFLD, thereby exhibiting potential therapeutic benefits in MetS-related disorders including MAFLD (131). Specifically, related data have identified new roles for leptin and adiponectin in controlling responses from MCs (133). MCs from the rat peritoneal cavity were found to express the receptors for both leptin and adiponectin and to respond to these adipokines by producing cytokines and reactive oxygen species. As might be expected, the responses to leptin and adiponectin are rather different. While both induce the migration of MCs, leptin induces the secretion of histamine and cysteinyl leukotriene, as well as the expression of CCL2, while adiponectin induces the production of anti-inflammatory IL-10. CCL2 is associated with the amount of MetS-related atherosclerotic lesions and lipid deposition in atherosclerotic plaques (134) and is one of the major chemokines that are involved in the development and progression of CVD (135). In contrast, adiponectin appears to offer potential protection against the development of MetS (136). Thus, these molecules play quite different roles in controlling the MCs-driven inflammatory responses. The identification of the molecular mechanisms through which adipokines exert their effects on MCs may provide the potential to developing therapeutic strategies for controlling these adipokine-mediated effects.

Regarding *H. pylori*-related MCs stimulation, this bacterium, by activating MCs, induces cytokines such as IL-33 and, by altering the gut microbiota and metabolic hormone concentrations, such as leptin, may also be involved in MetS-related disorders at the topical and systemic levels (56, 137, 138). Patients with *H. pylori* infection are characterized by significantly higher counts of MCs in the gastric mucosa compared to those of normal subjects, a finding accompanied by a high number of apoptotic cells as well (76).

The infiltration of MCs in the gastric epithelium is significantly higher for the *H. pylori* group, but their numbers are diminished after a successful eradication (139). A further *in vitro* study deduced that *H. pylori* enhances the release of histamine by murine serosal MCs (140). Moreover, the *H. pylori* neutrophil activating protein, which is regarded as a major virulence factor of the bacterium (141), activates, among others, MCs that secondarily secrete pro-inflammatory molecules (142). The aforementioned IL-33 induced by *H. pylori* has been shown to orchestrate MCs responses, favor bacterial expansion, and synergize to gastritis development (56). In addition, *H. pylori* stimulates MCs directly or *via* gastrin induction, and they are actively involved in the pathogenesis of *H. pylori*-associated pathologies. Beyond activated MCs, vascular endothelial growth factor (VEGF), IL-8, chymase or tryptase, and MCs growth factor are linked to *H. pylori* infection (38).

The intestine appears to be another site for *H. pylori* infection that can contribute to the pathophysiology of intestinal inflammatory disorders (143, 144). Beyond the upper gastrointestinal tract, *H. pylori* can colonize the intestine (145), indicating that *H. pylori* strains could adapt to colonizing extragastric niches such as the intestine and can trigger intestinal inflammatory disorders such as IBS (144, 146, 147) and IBD, at least in certain populations (143, 148–151).

Similarly, MCs are residents of the whole gastrointestinal mucosa, and their dysregulation has been implicated in the pathophysiology of several inflammatory disorders such as IBS and IBD (152, 153). In this regard, increased numbers of MCs can be located in the intestine of patients with IBD, as well as an augmented amount of MCs products (i.e., histamine and tryptase), and there is abundant evidence of MCs activation in the intestinal mucosa of patients with IBD. Moreover, MCs activation also contributes to the pathophysiology of IBS (152, 154). Increased concentrations of MCs mediators in the gastrointestinal tract have been detected in these patients, signifying a direct interaction between MCs and nerves in IBD and IBS (152). MCs products including histamine, proteases, prostaglandins, and cytokines could also contribute to hypersensitivity and intestinal mucosal permeability defects in patients with IBS (155). Importantly, both IBS and IBD are associated with CVD (156, 157).

Therefore, a potential direct interaction between active *H. pylori* infection and activated MCs taking place in the intestinal niche might contribute to the pathophysiology of intestinal inflammatory disorders such as IBS and IBD associated with CVD. Thus, further studies are needed to shed light on this critical interaction, thereby offering related important clinical implications.

In addition, MCs themselves can be stimulated by the corticotropin-releasing hormone, secreted under stress, to release mediators including histamine, tryptase, IL-8, and VEGF, which are involved in systemic pathologies (38). Moreover, *H. pylori*-induced VacA displays chemotactic activities to BMD-MCs and induces these BMD-MCs to produce pro-inflammatory cytokines, including TNF-α (52). *H. pylori* infection is connected to the increased serum concentrations of TNF-α, a circulating cytokine able to exert its effects at a distance. TNF-α and IL-6 play important roles in the regulation of the synthesis of other acute-phase proteins, which are recognized risk factors for MetS-related atherosclerosis (38). These cytokines also exhibit profound actions on lipid metabolism directly at the site of the atherosclerotic lesion and could influence the atheroma process *via* blood circulating concentrations, distant production of cytokines, or *via* stimulating the circulating white blood cells to produce them, thus contributing to the pathogenesis of MetS-related CVD and C-CVD (158). Furthermore, MCs are implicated in vascular dysfunction, a prominent feature of MetS (159, 160), and the release of vasoactive substances by activated MCs can bring about endothelial dysfunction and impaired vasodilation, promoting AH and atherosclerosis (159, 160). MCs-derived mediators, such as tryptase, can directly damage the blood vessels and exacerbate vascular inflammation (159, 161).

More specifically, MCs acting in the first line of defense against pathogens have been observed to promote the development and progression of atherosclerosis. Chronic inflammation is regarded as a pivotal player in atherosclerosis, and both *H. pylori* and MCs-driven inflammation converge to amplify this process (51, 162, 163). In individuals with MetS, the occurrence of *H. pylori* infection can further deteriorate systemic inflammation, creating a microenvironment conducive to endothelial dysfunction and lipid accumulation within the arterial walls (102). The combined effects of *H. pylori* infection and MCs activation on endothelial function may accelerate the development of atherosclerotic lesions. *H. pylori*-induced inflammation, coupled with MCs-mediated cytokine release, fosters a pro-inflammatory microenvironment within the arterial wall, favoring the recruitment of immune cells and the formation of atherosclerotic plaques (162–164). MCs are between the crucial immune effectors in MetS-related atheroprogression because they release not only the MCs-specific neutral proteases tryptase and chymase upon activation but also a whole array of mediators including histamines, cathepsins, growth factors, cytokines, and chemokines (165).

Experimental evidence has generally illuminated the crucial role of MCs in atheroprogression and plaque vulnerability (166). These results are in line with related investigations where MCs abundance is associated with symptomatic carotid artery disease (167) and the prevalence of future adverse events (168).

Importantly, genetic and pharmacological interventions targeting MCs activation have demonstrated promising results in ameliorating MetS-related pathologies in experimental models (162). This highlights the therapeutic potential of modulating the activity of MCs in the context of MetS.

Beyond atherosclerosis, the impact of *H. pylori* infection and MCs activation extends to vasculitis, a manifold group of inflammatory conditions characterized by blood vessel inflammation (169). Vasculitis can influence various vascular beds, including small- and medium-sized arteries, and its clinical manifestations vary, ranging from skin rashes to multi-organ involvement. *H. pylori* has been reported to accompany certain forms of vasculitis, particularly with dermal and renal ones (170, 171). The ability of this bacterium to trigger immune responses and promote smoldering chronic inflammation may induce or deteriorate vasculitis processes (172). Similarly, in patients with vasculitis, the occurrence of MetS appears to be an important predictor of all-cause mortality (173).

Furthermore, the activation of MCs, richly populated around blood vessels, plays a crucial role in the pathophysiology of vasculitis (116). MCs contribute to vasculitis through the release of vasoactive substances, such as histamine, that can directly damage blood vessels (161). In medical disorders such as cutaneous leukocytoclastic vasculitis, MCs activation and the subsequent histamine release may be implicated in the formation of characteristic skin lesions (174). The interaction between *H. pylori* infection, MCs activation, and vasculitis demands further investigation to shed light to the precise mechanisms and clinical implications.

Whereas atherosclerosis and vasculitis constitute prominent vascular diseases and are the common denominator to both *H. pylori* infection and MCs activation, it is crucial to acknowledge that the impact of these factors extends beyond these conditions. Vascular diseases encompass a wide spectrum of pathologies, including aneurysms, peripheral arterial disease, and thromboembolic disorders. For instance, the role of *H. pylori* infection in the development of abdominal aortic aneurysms has garnered attention (175, 176). Chronic inflammation induced by *H. pylori* may contribute to the degradation of the arterial wall integrity, thereby predisposing individuals to aneurysm formation (177). Furthermore, MCs have been proposed as key players in the formation and progression of thromboembolic events (178). The release of MCs-derived mediators, such as tissue factor and plasminogen activator inhibitor-1, can promote a prothrombotic state, enhancing the risk of thrombosis and embolism (178, 179). In this respect, *H. pylori* appears to stimulate the expression of plasminogen activator inhibitor-1, an important player in the pathogenesis of CVD (88). Understanding the intricate interplay between *H. pylori* infection, MCs activation, and thromboembolic disorders is a fertile ground for future research.

In summary, MCs possess a central position in the complex landscape of *H. pylori*-related MetS. Their activation within the adipose tissue contributes to chronic inflammation, IR, and immune cell recruitment. Moreover, MCs are key players in vascular dysfunction and atherosclerosis, a hallmark of MetS and *H. pylori* infection. Comprehending the precise mechanisms underlying the involvement of MCs in *H. pylori*-related MetS pathophysiology holds promise for innovative therapeutic interventions and potential clinical applications.

## 4 Impact of *H. pylori* infection and MetS-related MCs activation on CVD

**Figure 1:**
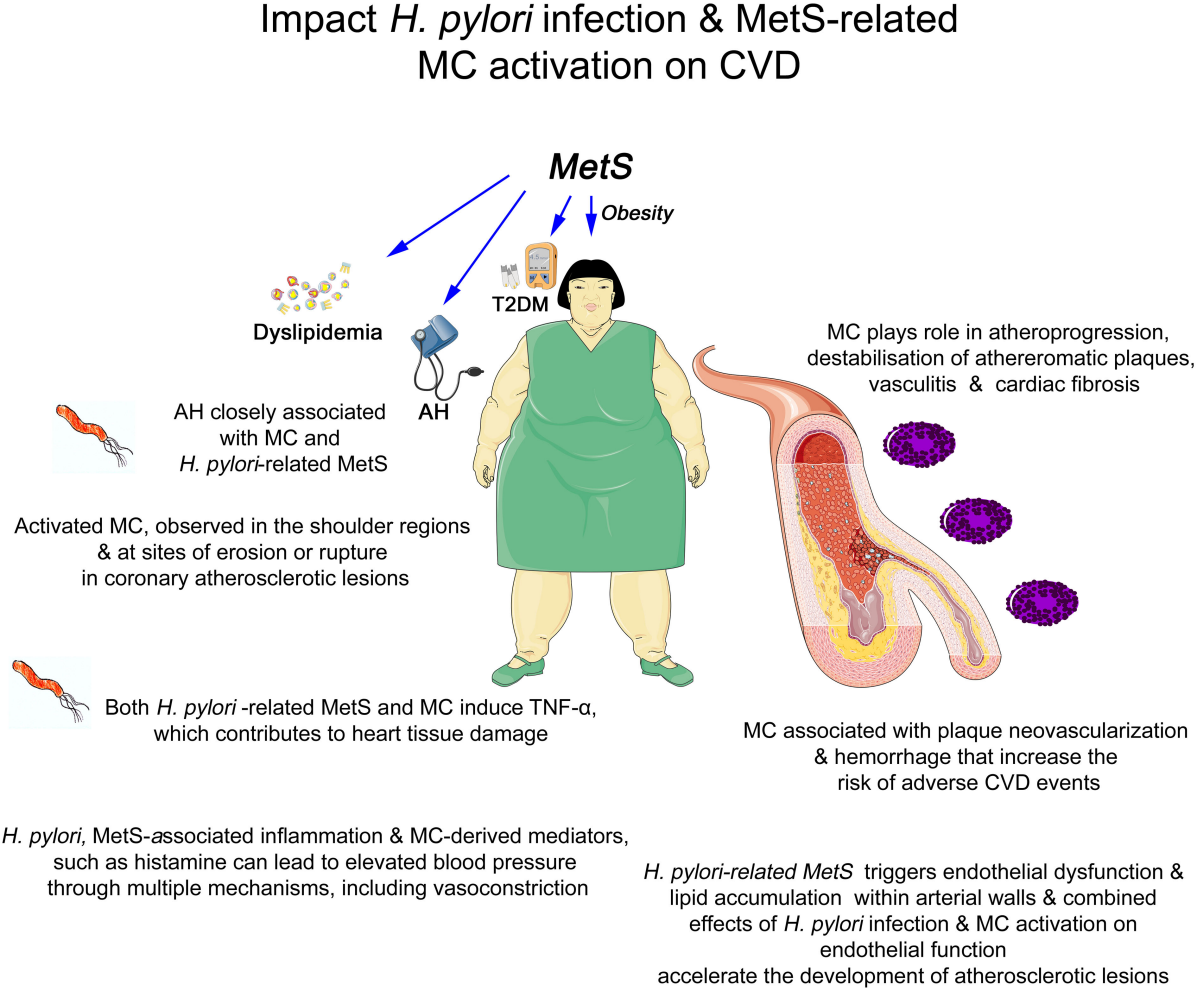
Graphical summary of the association between *Helicobacter pylori* infection, metabolic syndrome-related mast cell activation, and cardiovascular disorders. AH, arterial hypertension; CVD, cardiovascular disorders; MCs, mast cells; MetS, metabolic syndrome; T2DM, Type 2 Diabetes Mellitus; TNF-α, tumor necrosis factor alpha.

Activated MCs, which are associated with *H. pylori* infection and MetS, are frequently observed in the shoulder regions and particularly at the sites of erosion or rupture in coronary atherosclerotic lesions (**Figure 1**), i.e., in patients who died from myocardial infarction (180, 181). Moreover, human studies have shown an association of MCs with plaque neovascularization and hemorrhage that increase the risk of adverse CVD events (168). Furthermore, for instance, AH, which is prevalent in MetS and closely linked to both *H. pylori* infection and MCs activation, is associated with augmented premature morbidity and mortality due to CVD and stroke (182). Its high worldwide prevalence continues to increase and contributes to global morbidity and mortality, and as an important risk factor for *H. pylori* infection and MetS-related CVD, AH represents the principal cause of mortality related to *H. pylori* infection and MetS-associated ischemic heart disease and stroke (4). *H. pylori*-associated inflammation can lead to elevated blood pressure through multiple mechanisms, including compromised vascular function (4, 102). Similarly, MCs-derived mediators, such as histamine, can directly induce vasoconstriction, further exacerbating AH (183). In this respect, the MCs-revived tryptase induces cardiac fibrosis in the hypertensive heart (184). In addition, MCs may be implicated in hypertensive-related advance myocardial damage (185). Interestingly, activated MCs granules, beyond other mediators, also store and release the pro-inflammatory cytokine TNF-α, closely related to AH (186), which contributes to adverse myocardial remodeling by promoting cardiomyocytic apoptosis, hypertrophy, and inflammation. MCs degranulation is a significant contributor to inflammatory damaging processes (187), and in patients with MetS, TNF-α appears to be a CVD risk factor (188).

From a related clinical point of view, as the majority of hypertensive patients do not exhibit any symptoms, the disease is occasionally referred to as the “silent killer” and may be fatal due to cerebral strokes and ruptured arterial aneurysms. Heart disease, coronary artery disease, and stroke are just a few of the major issues caused by AH. Therefore, it is critical to understand high blood pressure risk factors and to take preventive measures (189).

As in the case of activated MCs-induced TNF-α that contributes to heart tissue damage, *H. pylori*-related MetS could also contribute to CVD by inducing pro-inflammatory cytokines, such as the aforementioned TNF-α (93). Beyond other mediators, *H pylori*-related increased circulating TNF-α, which is able to exert its effects at a distance, may also lead to heart tissue damage (158). Nevertheless, whether *H. pylori* and MetS-related MCs activation could also contribute to CVD progression by inducing pro-inflammatory mediators including TNF-α needs further investigation.

Existing related evidence indicates a central role for inflammatory cell-related processes, particularly MCs, in CVD (190). MCs accumulate excessively in the injured endothelium, which, by stimulating the formation of foam cells and atherosclerotic plaques and representing an important factor in plaque degradation and the initiation of coagulation due to the cytokines released from granules and proteolytic enzymes, plays a role in the pathogenesis of CVD (191). The MCs mediator, histamine, and its receptors profoundly impact the pathophysiology of the heart, leading to hypertension-induced cardiac hypertrophy and other cardiac disorders (190). Importantly, MCs degranulation occurs in cardiac injury (126). MCs degranulation increases rapidly and reaches to about 80% within a week after cardiac injury (192).

Especially, experimental studies in animals have clarified the critical role of MCs in atheroprogression and plaque vulnerability (166, 193). Chymase is linked to atherosclerotic plaque vulnerability (193). In humans, MCs have been shown to accumulate in advanced and ruptured coronary plaques (181). More recent data indicated that the connection of MCs with disease progression has been recognized in patients with CVD. Significantly augmented serum tryptase concentrations are detected in patients with acute coronary syndromes (168, 194), and the number of intraplaque MCs has been shown to increase upon atherosclerotic plaque destabilization (168). Equally, MCs in the human heart generate chymase and renin, which locally activate the angiotensin system and can cause arteriolar vasoconstriction, involved in the pathogenesis of CVD (195). Furthermore, an independent association of the number of intraplaque MCs in carotid plaques is connected to the prevalence of clinical CVD adverse events (168).

Finally, MCs are linked to cardiac fibrosis through several mechanisms (187). In this respect, for instance, MCs are able to produce various inflammatory cytokines including TNF-α, IL-6, amines (mostly histamine), and proteases, regulating the activity of matrix metalloproteases and collagen degradation, thus playing a role in the organization and resolution of inflammation and fibrosis in the myocardium (196). However, owing to the ability of MCs to induce both pro- and anti-fibrotic mediators, many studies have reported controversial results and defined MCs divergent functions, including negative, neutral, or protective activities of MCs in cardiac remodeling and fibrosis. Although preclinical and clinical data have identified MCs as important orchestrators of cardiac fibrosis, the exact mechanisms are yet to be clarified. Because MCs represent a valuable target for the therapeutic manipulation of fibrinogenesis, a better understanding of their involvement is required to stop and even reverse cardiac fibrosis.

In conclusion, the dual impact of *H. pylori* infection and MetS-related MCs activation extends to atherosclerosis, vasculitis, and a broader spectrum of vascular diseases. These interactions underscore the necessity for a comprehensive approach to understanding and managing vascular complications in individuals with MetS. Further research is essential to unraveling the intricate mechanisms driving these associations, potentially paving the way for innovative therapeutic strategies.

## 5 Translational insights for clinicians: reimagining diagnosis and treatment

The intricate interplay between *H. pylori* infection, MetS-related MCs activation, and their combined impact on CVDs presents clinicians with a unique opportunity to reimagine the diagnosis and treatment landscape. The multifaceted nature of these interactions proposes novel avenues for intervention, offering therefore a potential paradigm shift in managing patients at risk of CVD adverse events associated with *H. pylori*-related MetS.

One of the first translational insights lies in the identification of individuals at high risk of developing CVD within the context of both *H. pylori* infection and MetS. Recognizing that *H. pylori* infection may exacerbate the pro-inflammatory milieu in these patients, clinicians can consider routine testing for the presence of the bacterium. Although gastric histology for the detection of *H. pylori* represents the practical diagnostic gold standard for active *H. pylori* infection (197), noninvasive tests, such as the urea breath test or stool antigen test, can identify *H. pylori* infection with high sensitivity and specificity (197). In high-risk populations, such as those with MetS, routine screening may provide valuable insights into a patient’s overall risk profile.

Clinicians should consider not only the presence of *H. pylori* infection and MetS but also the immunological milieu, including markers of chronic inflammation and MCs activation accompanying these two disorders very frequent in routine clinical practice. Serum markers such as C-reactive protein, IL-6, IL-17, TNF-α, and/or IL-22 can serve as indicators of smoldering chronic inflammatory processes with concomitant MCs activation (198, 199). Increased levels of such markers, particularly in individuals with MetS and allergic and inflammatory disorders (199–201), may prompt further investigation into the potential involvement of *H. pylori* infection and MCs activation. In addition, the measurement of MCs-specific mediators, such as histamine, chymase, and tryptase, may offer insights into the extent of MCs activation (161, 201). While these tests are not routine in clinical practice, they represent a promising avenue for future research and development. Addressing these factors may complement the existing treatment approaches for MetS, thereby improving patient outcomes. In patients with both *H. pylori* infection and MetS, therapy to eradicate *H. pylori* could be considered as an adjunct to standard treatment (202, 203). Moreover, it may synergistically contribute to mitigate the inflammatory burden associated with *H. pylori* infection (6), potentially alleviating some of the systemic effects attributed to MetS and CVD (158, 204).

Regarding the impact of probiotics on the eradication of *H. pylori* and consequence of CVD, for more than 15 years, it has been well established that eradication regimens supplemented with probiotics increase the rates of eradication and reduces the side effects in *H. pylori* infection (205–208). The incorporation of probiotics into complex eradication therapy holds promise for optimizing the treatment of *H. pylori* infection. Probiotic strains, through their alteration of the gastric and gut microbiota, immunomodulatory properties, and their direct antagonistic effects against *H. pylori* (*via* bacteriocins and other factors such as bacterial synthesized acids and hydrogen peroxide), can increase the eradication rates, decrease the frequency and severity of side effects, and enhance patient compliance and treatment outcomes.

Apart from gastrointestinal pathologies, the impact of probiotic supplementation has been largely reported to reduce the risk of systemic pathologies, including cardiometabolic syndrome, and increase the *H. pylori* eradication rates (209). Supplementation of next-generation probiotics has been reported to reduce IR, plasma cholesterol, and the risk of developing liver dysfunction and inflammation, whereas no significant change is demonstrated in the gut microbiota (210). Probiotic supplementation can improve the clinical parameters of NAFLD, the hepatic component of MetS, and the eradication rates of *H. pylori* (211). Similarly, prebiotics and/or probiotics can exert a beneficial effect in the prevention of *H. pylori*-related neurodegenerative disorders (212).

Beyond MCs stabilization, MCs silencers constitute novel therapeutic approaches with translational potential (158, 162, 204, 213, 214). These agents could be evaluated as adjunctive therapies for individuals with *H. pylori* infection and MetS, particularly those with evidence of MCs activation-associated CVD adverse events.

Translational insights should also inform the education and lifestyle modification strategies that offer benefits to patients with MetS (215). Moreover, lifestyle interventions appear to decrease the risk of *H. pylori*-related gastric cancer prevalence and mortality (216). Of note is that *H. pylori* infection with MetS and/or MCs appears to be involved in gastrointestinal cancer pathophysiology (217–219) and metastases (217, 218, 220).

Emphasizing the potential impact of *H. pylori* infection and MetS-related MCs activation on CVDs can motivate patients to adhere to lifestyle interventions, i.e., dietary and exercise recommendations. Highlighting the potential role of infectious and immunological factors in the development of CVDs can enhance patient engagement and compliance with these interventions.

Translational insights are not static: they pave the way for future research directions and clinical advancements. Further investigations are warranted to elucidate the exact mechanisms linking *H. pylori* infection, MCs activation, and CVD within the context of MetS. Longitudinal studies should explore the impact of *H. pylori* eradication and MCs stabilization therapies and/or silencer novel therapeutic approaches on the clinical outcomes of individuals with MetS. These studies can help determine the effectiveness and safety of these interventions, providing valuable guidance for clinical practice. Biomarkers that reflect MCs activity and inflammation could enhance risk stratification and guide personalized treatment strategies. As mentioned earlier, very recent data have indicated that the oral cavity is an additional niche for *H. pylori* and could be the trigger of *H. pylori* infection, re-infection, and transmission into the stomach (77). Thus, further research is expected to elucidate the potential direct interaction between *H. pylori* and MCs taking place in the oral cavity, with a potential relation to CVD. In this respect, apart from *H. pylori*-related dysbiosis in the stomach, intestine, the ocular surface, and intraocular cavity, oral cavity *H. pylori*-related dysbiosis can also contribute to the pathophysiology of MetS component-associated glaucoma, CVD, and neurodegeneration (221, 222). Therefore, targeting such dysbiosis could be important to the management of glaucoma, CVD, and other neurodegenerative disorders (221, 222).

All in all, the translational insights derived from understanding the intricate crosstalk among *H. pylor*i infection, MCs activation, MetS, and CVD offer a new era for clinicians. Identifying high-risk patients, adopting integrated diagnostic approaches, and tailoring therapeutic strategies represent tangible steps toward enhanced patient care. These insights not only improve our understanding of the pathophysiology of MetS but also permit innovative approaches that may ultimately lessen the impact of CVD in patients with MetS.

## Author contributions

MD: Investigation, Visualization, Writing – original draft, Writing – review & editing. AP: Investigation, Validation, Writing – review & editing. SP: Investigation, Writing – review & editing. MB: Investigation, Writing – review & editing. EK: Investigation, Writing – review & editing. MT-C: Investigation, Writing – review & editing. EV: Investigation, Writing – review & editing. CH: Investigation, Writing – review & editing. HK: Investigation, Writing – review & editing. DR: Investigation, Writing – review & editing. CK: Investigation, Writing – review & editing. IL: Investigation, Writing – review & editing. MT: Investigation, Writing – review & editing. AS: Investigation, Writing – review & editing. JK: Conceptualization, Investigation, Project administration, Supervision, Validation, Writing – original draft, Writing – review & editing.
